# Supplementary material for: Quasi-spectral characterization of intracellular regions in bright-field light microscopy images
Source: Sci Rep. 2020 Oct 27;10:18346. doi: 10.1038/s41598-020-75441-7 (PMC7591573; doi:10.1038/s41598-020-75441-7)
Supplement: Supplementary file 1 — Supplementary Information. [file 41598_2020_75441_MOESM1_ESM.pdf]

Quasi-spectral characterization of intracellular regions in bright-field light microscopy images

Kirill Lonhus, Renata Rychtáriková, Ganna Platonova & Dalibor Štys

1 SIMULTANEOUS CALIBRATION OF DIGITAL CAMERA AND OPTICAL PATH

The calibration of the optical path and camera chip together with image (raw files) correction was performed in the following steps:

1. Experimental Part:
   a. Using a microscope photocamera, images (raw files) of the set of gray layers covering a 2 mm thick glass (type Step ND Filter NDL–10S–4) on a microscope stage were captured successively. The image of zero and the highest intensity was acquired at zero photon flux reaching the camera chip (at least, we were trying to take a picture only with response of camera noise) and without any layer, respectively. The image of each gray layer was taken at least in 6 parallels. A mean calibration image was computed as an intensity average for each pixel through all parallel measurement. The microscope and microscope camera was arranged and set up the same as in the biological experiment.
   b. The microscope objective was replaced for a fibre spectrophotometer Ocean Optics USB 4000 VIS-NIR-ES by which the spectra (Fig. 3a in the main text) of the series of the gray layers (including the zero and the highest spectrum) relevant to the gray images were measured successively. The spectrum of each gray layer was taken at least in 6 parallels from which a mean spectrum was obtained.
2. Computational Part:
   a. Construction of a calibration curve for each pixel
      (1) Red, green, and blue camera Bayer filter profiles (supplied by a camera producer; Fig. 3b) were transformed into the numeric format. The results are saved in the spectrum.txt file of the calibration data.
      (2) The light spectra captured by each pixel of the colour camera filter were obtained by multiplication of the measured incident spectra by the digitalized camera filter.
      (3) For each gray layer, a total number of photons (i.e., counts) captured by each pixel was calculated as an integral (trapezoidal rule) of the area below the respective incident spectrum (Fig. 3c).
      (4) For each pixel of the mean calibration image (see item 1a), a calibration point was constructed (Fig. 3d) as a dependency of the total number of photons reaching the pixel on the pixel intensity. Each pair of two consecutive calibration points was fitted by linear interpolation.
   b. Intensity correction of the series raw file
      (1) Using the calibration relation of the relevant section of the calibration curve (Fig. 3d), the intensity of each pixel of the testing image (i.e., raw file of the L929 cell) was converted to values that can, after the calibration of the fibre spectrophotometer, correspond to the total number of photons (in double precision numbers).
      (2) For further image operations, the resulted matrix was transferred into a 14-bit PNG format.

The preparation of the calibration curve (item 2a) is the content of Algorithm 1. The series raw files were then corrected using Algorithm 2 described in item 2b. Both algorithms are implemented in the VerCa software (Institute of Complex Systems, Nové Hrady, CZ).

Input: **sQE** as a quantum efficiency spectrum of one colour camera channel;
sFt as a spectrum of a stack of gray layers (N-item folder);
iFt as a relevant colour channel of the stack of gray layers (N-item folder);
Output: **k** as a matrix of the slopes of the linear sections of the calibration curve;
s as a matrix of the shifts of the linear sections of the calibration curve;
int as a matrix of the intensities which demarcates the ranges of the linear sections of the calibration curve;

A = zeros(N,1);   *% create an empty (zero) N-element vector*

for $i = 1$ **to** N **do**
  sFt = readSp(i);
    % read a spectrum **sFt** *for (i) gray layers*
  wSp = **sFlt** .* **sQE**;
    % for each wavelength, weight the spectrum **sFt** *by the spectrum* **sQE**
  A(i) = integrateSpectrum(**wSp**);
    % integrate the area under the weighted spectrum to obtain a total number of photons reaching the colour channel of the camera chip
end

int = zeros(N, nPx);
k = zeros(N, nPx);
s = zeros(N, nPx);
  % create empty (zero) matrices of the output calibration files (i.e., of the calibration parameters)

for $i = 1$ **to** $N - 1$ **do**
  iFt1 = readIm(i) ;
  iFt2 = readIm($i + 1$) ;
    % read raw image files of relevant colour channel for (i) and (i+1) gray layers

  for $j = 1$ **to** nPx **do**
    int(i, j) = **iFt1**(j);
      % read and save the first edge point of the section of the calibration curve
    k(i, j) = (**A**($i + 1$) - **A**(i))/(**iFt2**(j)- **iFt1**(j));
    s(i, j) = **A**(i)-**k**(j) .* **iFt1**(j);
      % calculate and save a slope and a shift of the relevant section of the calibration curve
  end
end

Algorithm 1: Construction of the calibration curve and creation of the calibration file for one colour channel.

2 METHOD VALIDATION

In order to ensure that the method is sensitive to composition, not only to the thickness, simple phantom experiments were conducted. In each of experiment, we recorded an image containing the border between

Input: I as an original (nPx)-resolved uncorrected raw image file;
int, **k**, and **s** as a content of the calibration file (see Algorithm 1)
Output: cI as a corrected image

cI = I .* 0;   *% create an empty (zero) matrix of the size of the* **I**

for $j = 1$ **to** nPx **do**
  if $\boldsymbol{I}(j) < \boldsymbol{int}(2, j)$ **then**
    cI(j) = **k**(1,j) .* **I**(1,j) + **s**(1,j);

  else if $\boldsymbol{I}(j) \in \langle \boldsymbol{int}(2, j), \boldsymbol{int}(3, j))$ **then**
    cI(j) = **k**(2,j) .* **I**(2,j) + **s**(2,j);

  else if $\boldsymbol{I}(j) \in \langle \boldsymbol{int}(3, j), \boldsymbol{int}(4, j))$ **then**
    cI(j) = **k**(3,j) .* **I**(3,j) + **s**(3,j);
    ...

  else
    cI(j) = **k**(N,j) .* **I**(N,j) + **s**(N,j);
  end
end
  % for intensity of each pixel of the image **I**, *find the relevant linear section of the calibration curve and use its mathematical relation to recalculate this intensity to the total number of photons*

Algorithm 2: Image correction of one colour channel.

two known substances (Fig. S1). In the first experiment, we investigated egg protein-air interface (**a**); in the second experiment, we dealt with oil-air interface (**b**). The aim of the phantom experiment was to test ability of the proposed method to enhance unsupervised clusterization. We applied the k-means algorithm (cosine distance, 1000 iterations, 6 clusters) to the original data and obtained relevant spectra (Fig. S1). The cosine distance was chosen, as it is one of the best method for feature comparison and performs in the RGB space well. One can see a more distinct separation of the raw data (**a, b**) and their quasi-spectral reconstructions (**c, d**).

In order to numerically prove the observation, we used a gap statistics criterion, which robustly represents the accuracy of the clusterization (see Tibshirani R. et al., *J. R. Stat. Soc.* B 63(2), 2001). We performed 128 repetitions of the clusterization with different random seeds. The values of criteria for raw RGB data and the relevant, acquired, quasi-spectra, respectively, are shown in Fig. S1(**e**). In both cases, the quasi-spectra show higher values of criteria and, thus, they are clusterized better. Also, the dispersion of the quasi-spectral clustering is less than the dispersion of the raw values. This indicates a greater stability of the proposed quasi-spectral method.

In order to check the method stability and consistency, we independently applied the method to two images (raw files) of the different cells from the same culture of L929 cell line (Fig. S2). The obtained mean quasi-spectra are quite similar, but not exactly — the cells may have a similar composition but they definitely have different shapes and thicknesses.

Figure S3 shows an example of non-monotonous dependencies of the cost and the variation coefficient, respectively, in optimization of the computation process of the microscopy image quasi-spectra.

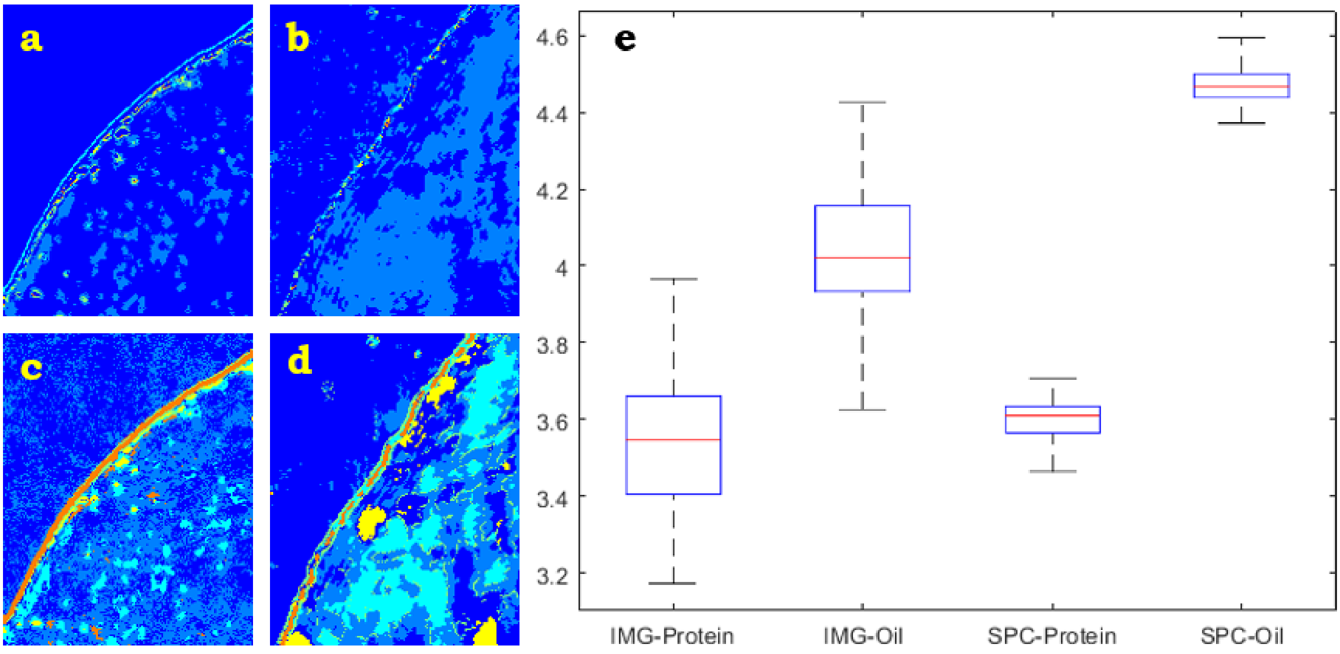

Figure S1. Unsupervised clusterization of images by k-means ($n = 8$, cosine metric) for egg protein-air interface **(a)** and oil-air **(b)**. The corresponding clusterization of the spectra is in **(c, d)**. The distribution of the gap criteria is shown in **(e)**.

3 SEMANTIC SEGMENTATION BY CONVOLUTION NETWORK

To prove the merit of the method of quasi-spectra estimation in semantic segmentation, we performed a well-known convolution neural network (CNN) – U-Net – on the original calibrated raw (ordinary) microscopy images, the contrast-enhanced images (after saturation of 1% of the lowest and highest intensities and stretching the remaining intensities to the original intensity interval accordingly), and the spectral images reconstructed by the novel method. The U-Net network (e.g., Ronneberger O. et al., *MICCAI 2015*, 2015) was designed for medical image segmentation. It is a classical encoder-decoder network with skip-layer connections. Thus, this CNN can achieve a pixel-wise accuracy. The U-Net network is well balanced and performs well in practice. More importantly, this CNN can be trained with very few images with intensive augmentation. (Most of the semantic segmentation networks require hundreds of images even with augmentation.)

The computations (100 epochs, approx. few hours per dataset) were conducted with a usage of a classical U-Net (see the main text), Tensorflow 2.1, and Python 3.6.10. on NVIDIA TI 1080 GPU. We used only 6 images for training and 1 image for validation. To mitigate possible overfitting, intensive dropping out in layers (0.5) was placed after each convolution layer, except the first one. Also, the image augmentation operations, executed consecutively as follows, was employed:

a) horizontal or vertical image flipping with chance 0.5,
b) elastic deformation: α = [0..40], σ = [4..8],
c) affine transformation: rotation to [-70..+70]°, translation to [-50..+50]%, scaling [0.5..1.5],
d) the Gaussian blurring, σ = [0..3],
e) the Gaussian noise, scale = [0..0.1],

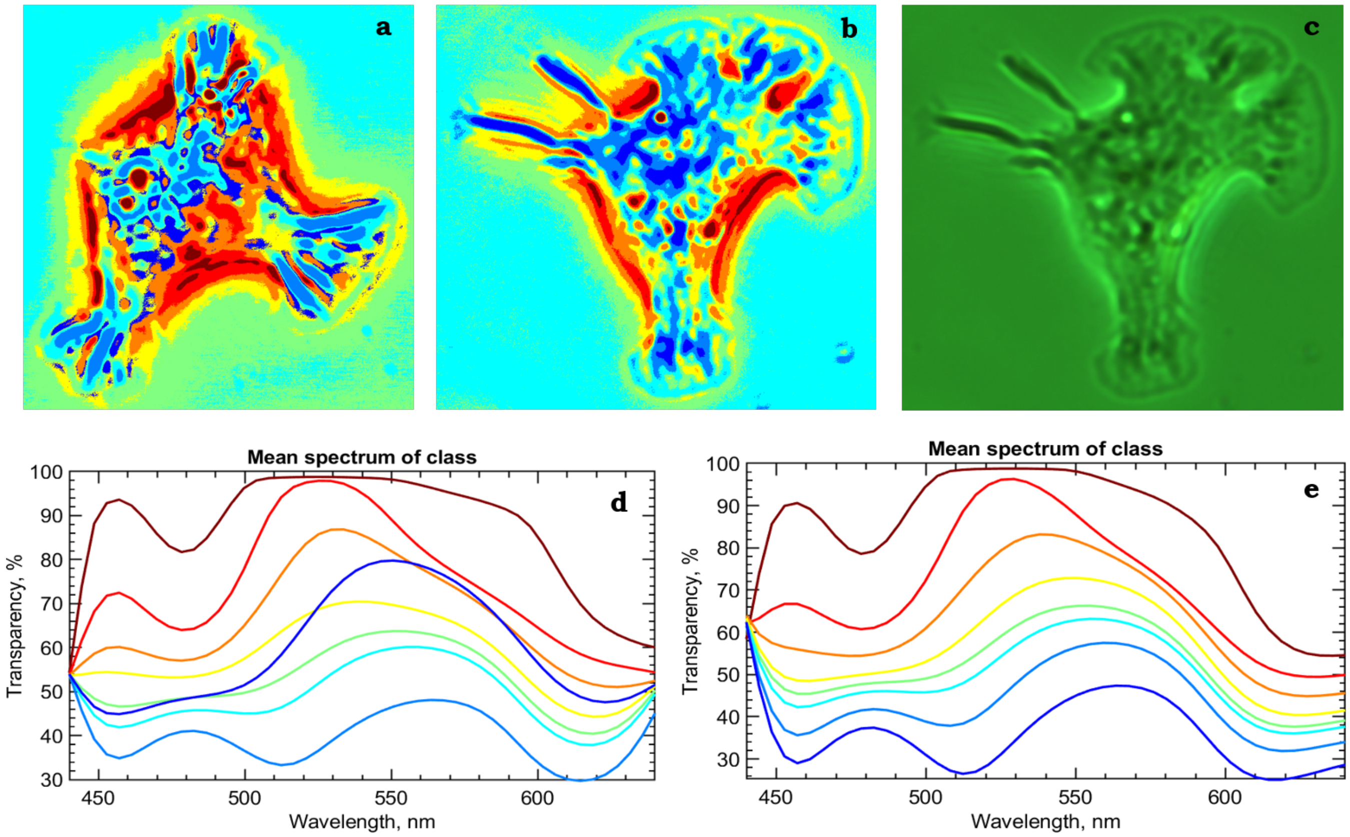

Figure S2. Independent clusterization (k-means, $n = 8$), cosine metrics) for two different fixed cells of L929 (**a**, **b**) and the corresponding spectra (**d**, **e**). The mean spectra of the corresponding clusters are quite similar, despite being obtained from completely different images and objects. Image **c** is original to spectral image **b**.

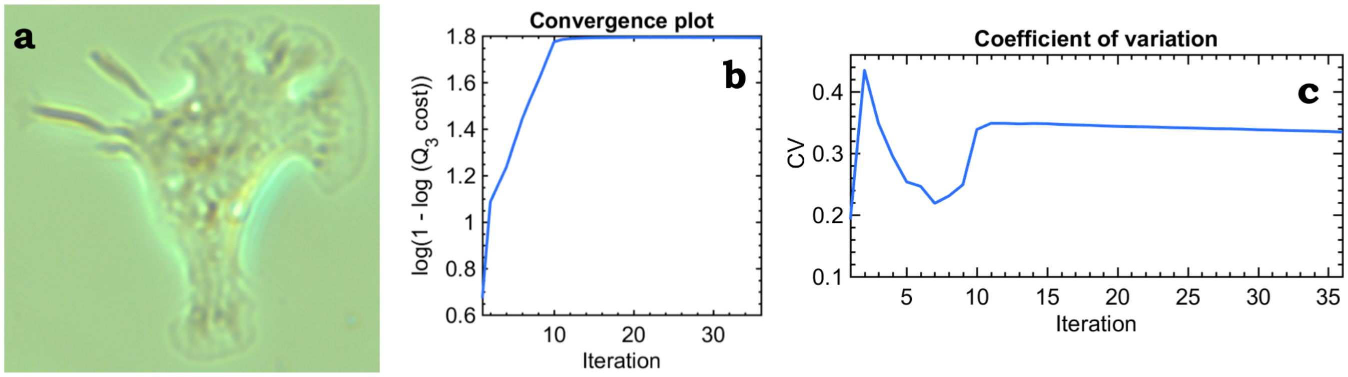

Figure S3. A raw image of a fixed cell of L929 from the bright-field light microscope combined with the effective light spectra was used for optimization, convergence curve (**b**). The variation coefficient (**c**) shows self-organization of the model. After reconstruction of the transparency spectra, the image can be viewed under arbitrary illumination such as the absolute black body with T = 5800 K, see (**a**).

f) dropout, chance = [0..0.05].

From the specified intervals listed above, the exact values of the parameters were drawn as uniform random values. In the list above, the elastic deformation is the most crucial operation and usually a very

powerful tool for increasing learning efficacy of semantic segmentation networks. In this case, a random displacement field was created, smoothed, and used for warping the image.

Each image of the triplet (ordinary, enhanced, and spectral) was derived from a single (the ordinary) image and, thus, the comparison of the image segmentation quality was valid for a small number of images such as this set is. Even though designed for ordinary images and not for hyperspectral-like images, the network architecture was not changed.

The results of the segmentation analysis can be seen in Fig. 4f (in the main text). As a measure of quality of the segmentation, we used the Intersection over Union (IoU) that is a classical measure for the semantic segmentation accuracy. As seen in Fig. 4f, the new quasi-spectral approach increased ($> 10\%$) the segmentation accuracy (IoU) substantially and converged much faster at achieving the maximal performance at the 8th epoch, while the contrast-enhanced image achieved only 80% accuracy (vs. 90% for quasi-spectral image) not until 40 epochs. The quasi-spectral images were much ($32\times$) larger than the ordinary images which made the learning process slower (about 30% per epoch). However, despite this fact, the quasi-spectral images showed a much faster segmentation process due to a fast convergence of the results (expressed as a dependence of the IoU on the number of the computational epochs).
